# A First Insight into a Draft Genome of Silver Sillago (*Sillago sihama*) via Genome Survey Sequencing

**DOI:** 10.3390/ani9100756

**Published:** 2019-10-01

**Authors:** Zhiyuan Li, Changxu Tian, Yang Huang, Xinghua Lin, Yaorong Wang, Dongneng Jiang, Chunhua Zhu, Huapu Chen, Guangli Li

**Affiliations:** 1 Guangdong Research Center on Reproductive Control and Breeding Technology of Indigenous Valuable Fish Species, Fisheries College, Guangdong Ocean University, Zhanjiang 524088, China; 2 Southern Marine Science and Engineering Guangdong Laboratory, Zhanjiang 524025, China

**Keywords:** *Sillago sihama*, Genome size, Guanine and Cytosine (GC) content, simple sequence repeat (SSR)

## Abstract

**Simple Summary:**

Silver sillago (*Sillago sihama* Forsskål) is distributed alongshore from the Indian Ocean to the West Pacific. Owing to its delicate quality, rich seafood taste, and high nutritional value, *S. sihama* is an attractive seafood in China. However, the main supply of this fish is from wild capture. The lack of genetic and genomic data for *S. sihama* has led to limited improvement in its breeding programs. In this study, we conducted a genomic survey of *S. sihama* using next-generation sequencing technology to investigate its genomic profile. We obtained useful data, such as genome size, sequence repeat ratio, heterozygosity ratio, and the genome sequences, which might accelerate the breeding and culturing programs for *S. sihama*.

**Abstract:**

*Sillago sihama* has high economic value and is one of the most attractive aquaculture species in China. Despite its economic importance, studies of its genome have barely been performed. In this study, we conducted a first genomic survey of *S. sihama* using next-generation sequencing (NGS). In total, 45.063 Gb of high-quality sequence data were obtained. For the 17-mer frequency distribution, the genome size was estimated to be 508.50 Mb. The sequence repeat ratio was calculated to be 21.25%, and the heterozygosity ratio was 0.92%. Reads were assembled into 1,009,363 contigs, with a N50 length of 1362 bp, and then into 814,219 scaffolds, with a N50 length of 2173 bp. The average Guanine and Cytosine (GC) content was 45.04%. Dinucleotide repeats (56.55%) were the dominant form of simple sequence repeats (SSR).

## 1. Introduction

Silver sillago (*Sillago sihama* Forsskål) is distributed alongshore from the Indian Ocean to the West Pacific [1]. When adult sillago are scared, they bury themselves in the sand [2]. Polychaete worms, amphipods, small prawns (*Penaeus*), and shrimps are the main source of food for sillago [3]. This fish is found along the southern seashore of China [4]. Owing to its delicate quality, rich seafood taste, and high nutritional value [5], *S. sihama* is an attractive seafood in China. However, the main supply of this fish is from wild capture [6]. Studies of this species have mainly focused on salinity tolerance [7], population dynamics [8], distinction of the genus *Sillago* [9], and phylogenetic relationships among the genus *Sillago* [10]. These studies found that *S. sihama* had tolerance to lower salinities [7], but with excessive exploitation, wild populations of *S. sihama* had been diminished in size and were low-aged [8]. The lack of genetic and genomic data for *S. sihama* has led to limited improvement in its breeding programs [11]. It is necessary to study the genome size and genome characteristics for *S. sihama*, which will provide genetic and genomic resources.

High-throughput next-generation sequencing (NGS) is currently the main approach for genomic surveys and is an important and efficient strategy for generating genetic and genomic information [12,13,14,15]. A genomic survey can boost progress in gene finding and phylogenetic analysis, and in understanding genetic variety, genome structure, and genetic improvement of advantageous characteristics [16,17,18,19], which could also accelerate breeding and culturing progress of *S. sihama*.

## 2. Materials and Methods

### 2.1. Specimen Materials

Specimens of *S. sihama* were obtained from Guangdong Ocean University Breeding Base. Two *S. sihama* specimens, named specimen 1 (female) and specimen 2 (male), were subjected to genome sequencing. All animal experiments were conducted in accordance with the guidelines and approval of the Animal Research and Ethics Committees of the Institute of Aquatic Economic Animals of Guangdong Ocean University (201903003).

### 2.2. DNA Extraction, Library Construction, and Sequencing

Genomic DNA was extracted from a *S. sihama* muscle sample using the SDS (sodium dodecyl sulfate) method [20] and randomly fragmented using a Covaris ultrasonic shearing device. Fragments with a length of ~350 bp were used to construct two paired-end DNA libraries, and then sequenced using the Illumina HiSeq X Ten platform with a read length of 2 × 150 bp, following the manufacturer’s protocol. After reads containing adapters or contaminations and low-quality reads were removed, clean reads underlying all following analyses were acquired. Entire read sets were deposited in the Short Read Archive (SRA) databank (http://www.ncbi.nlm.nih.gov/sra/), and are available under the accession number PRJNA545388.

### 2.3. Genome Size Estimation and Identification of Heterozygosity Ratio and Repeat Ratio

An estimate for the genome size of *S. sihama* was based on the K-mer frequency of the clean reads (k = 17) and the 17-mer frequency (depth) distribution was consistent with the Poisson distribution. From the distribution of 17-mer depth, we acquired the peak depth value, which represents the average value and variation of the related Poisson distribution [21,22]. Calculation of K-mer depth distribution for clean sequence reads and estimation of genome size were performed via Jellyfish (v2.2.4) software [23]. Because K-mer depth distribution can be affected by heterozygosity and repetitive sequences in the genome, the revision of genome size was performed. We also inferred the heterozygous frequency and repeat frequency based on K-mer analysis.

### 2.4. Sequence Assembly and Analysis of Guanine and Cytosine (GC) Content

Genome sequence assembly was performed using the de Bruijn graph algorithm available in SOAPdenovo (v2.04) [24,25]. Contigs were realigned using all clean reads and scaffolds were constructed step by step using diversified insert size paired-ends [26]. A K-mer size of 41 was set as the default assembly parameter. GC content along the assembled sequence was calculated from the proportion of GC out of the total number of bases in the sequencing data [27].

### 2.5. Identification of Simple Sequence Repeats (SSRs)

In order to identify simple sequence repeat (SSR) markers, SSRs were searched in the assembled scaffolds using SR search software [28]. The minimum base number for SSR identification of di-, tri-, tetra-, penta-, and hexa-nucleotides was 12 [29].

## 3. Results

### 3.1. Genome Sequencing and Sequence Quality Estimation

The 350 bp insert libraries were sequenced and a total 54.837 Gb (specimen 1)/54.452 Gb (specmen-2) of raw reads was generated (Table 1). After filtering and correction, a total of 45.063 Gb (specimen 1)/38.583 Gb (specimen 2) of clean reads were derived, with an error rate of approximately 0.03% for both samples. The Q20 values were both above 95%, while the Q30 values were both above 90%. Here, 5000 random clean reads from each specimen were used as a query sequence with BLAST (The Basic Local Alignment Search Tool) against the Nucleotide Sequence Database from NCBI (National Center for Biotechnology Information), and the result showed that there was no contamination from other species (Table S1). We present specimen 1 in the main text and specimen 2 in the Supplementary Materials, because differences in survey data between the two specimens were very small.

### 3.2. Genome Size, Ratio of Heterozygosity and Repeats

**Figure 1:**
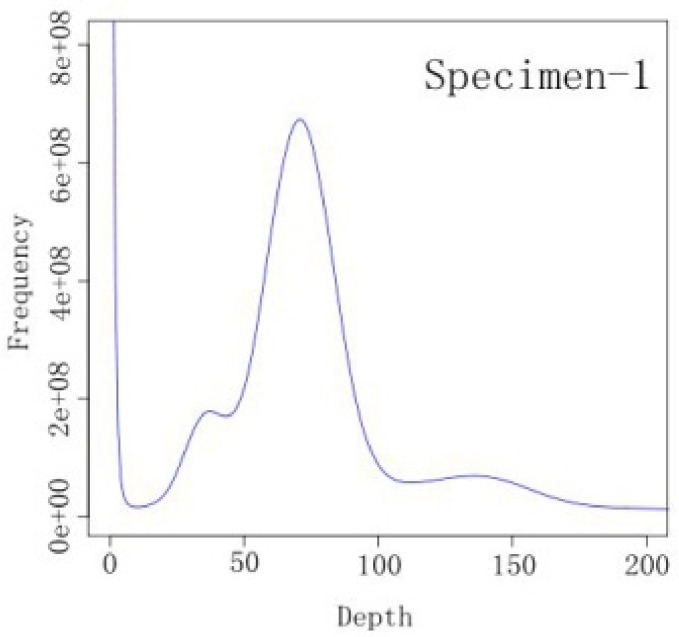
K-mer (k = 17) analysis for estimation of the genome size of *S. sihama* (specimen 1).

K-mer analysis was performed on all of the clean data. For the 17-mer frequency distribution (Figure 1, specimen 2 in Figure S1), the number of K-mers was 36,648,430,961 and the peak depth distribution was set at 70×. The estimated genome size was 523.55 Mb, which was calculated via the following formula:

Genome size = K−mer num/Peak depth(1)

which was based on the output of Jellyfish (v2.2.4) [23]. Then, the genome size was revised by excluding the K-mer error, via the following formula:

Revised genome size = Genome size × (1−Error Rate),(2)

giving a revised genome size of 508.50 Mb. The genome sequence repeat ratio percentage for *S. sihama* was 21.25% and the proportion of heterozygotes was 0.92% (Table 2, specimen 2 in Table S2).

### 3.3. Genome Assembly

With 41 bp K-mers, de novo assembly was performed using all of the clean reads. A total of 568,556,466 bp scaffolds were derived, with a N50 scaffold value of 2173 bp (Table 3, specimen 2 in Table S3). The N50 / N90 of the contigs / scaffolds were derived by ordering all sequences, adding all the contigs / scaffolds from the longest to the shortest and when the added length reached 50% / 90% of the total length of all contigs / scaffolds, the length of the last added contig / scaffold was the N50 / N90 [15].

### 3.4. GC Content

**Figure 2:**
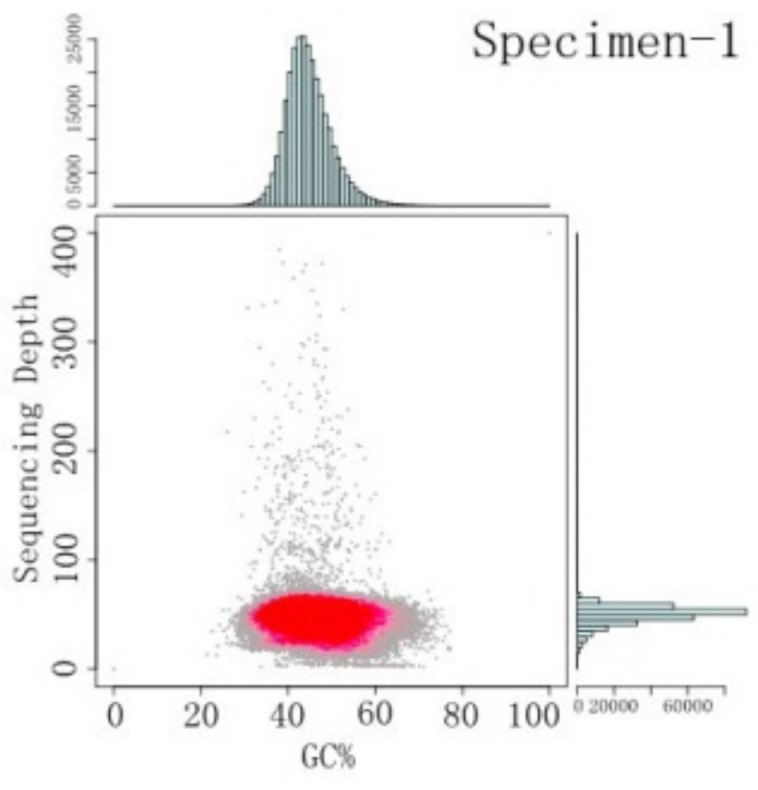
GC content and average sequencing depth of *S. sihama* (specimen 1) genome data used for assembly. For the spot graphs, the x-axis is GC content and the y-axis is sequencing depth. For the bar graphs, the x-axis is sequencing depth distribution and the y-axis is GC content distribution.

The GC content of the *S. sihama* genome and average sequencing depth were plotted along the assembled sequence (Figure 2, specimen 2 in Figure S2). The density points were only concentrated in the 30–65% range, and the average GC content was 45.04%.

### 3.5. Identification of SSR

**Figure 3:**
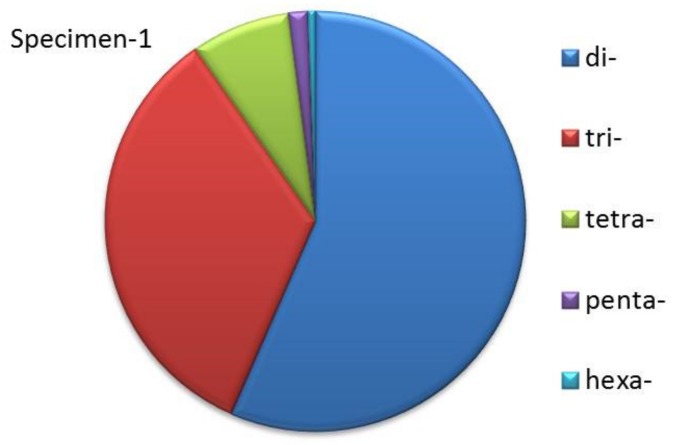
Ratio of different SSRs in *S. sihama* (specimen 1).

The total number of identified SSRs was 149,257 (Table 4, specimen 2 in Table S4). Dinucleotide repeats were dominant (56.55%), followed by trinucleotide repeats (33.78%), tetranucleotides repeats (7.61%), pentanucleotide repeats (1.47%), and hexanucleotide repeats (0.58%) (Figure 3, specimen 2 in Figure S3).

## 4. Discussion

In recent years, with the development of NGS technology [30], efficient approaches, such as faster sequencing, longer reads, and cost reduction [31], have been provided for researchers to cope with a wide range of questions from newly-found and non-model species, such as *Procambarus clarkii* [19], *Sillago sinica* [4], and *Pelteobagrus fulvidraco* [32]. Moreover, the estimation of genome size by the K-mer method using genome survey sequences makes genome size estimation available for non-model species, without any prior knowledge [15]. According to the K-mer (k = 17) analysis, the genome size of *S. sihama* was ~508.50 Mb. The genome size of *S. sihama* was close to the size of *S. sinica* (534 Mb) [4] and *Gambusia affinis* (598.7 Mb) [33], but smaller than *Oryzias latipes* (700.4 Mb) [34], *P. fulvidraco* (714 Mb) [32], and *Oreochromis niloticus* (1.082 Gbp) [35]. The genome size of *Sillaginidae* is relatively small, as a result of lower number of repetitive sequences in the *Sillaginidae* genome [4].

For the genome assembly, if the heterozygosity rate is higher than 0.5%, it is difficult to assemble, and if higher than 1%, it is even more difficult [23]. We found that the heterozygosity rate of *S. sihama* was ~0.92%. The repeat rate of *S. sihama* genomic sequences was ~21.25%. The characteristics of the *S. sihama* genome might impact the accuracy of genome size estimation. This was the reason that revision of genome size was performed. Before the appearance of a more efficient de novo assembly method, a reference genome was necessary for a good genome assembly [19].

In our study, the N50 scaffold value was 2173 bp and the N50 contig value was 1362 bp (Table 3). As de novo assemblies obtained from NGS technologies are delicate debris, a good genome assembly requires N50 contigs > 30 kb and N50 scaffolds > 250 kb [36]. However, a reference genome should be available to map short read sequences to a good genome assembly [19]. Our study was a first draft genome and stands as a useful reference for further studies on whole genome sequencing of *S. sihama*.

## 5. Conclusions

In this study, the first reference genome of *S. sihama* was presented. The genome size of *S. sihama* was ~508.50 Mb, with 814,219 scaffolds and a N50 length of 2173 bp. The genome sizes of *S. sihama* were close to *S. sinica* (534 Mb), which shared a very close relationship with *S. sihama* during evolution [4], indicating that the result of this study was credible. Regarding the N50 values for contigs and scaffolds in this study, there are still improvements to be made in the research of the genome of *S. sihama*.

## Figures and Tables

**Table 1:** Statistics of *S. sihama* genome sequencing data.

Library	Insert Size (bp)	Raw Base (bp)	Effective Rate (%)	Clean Base (bp)	Error Rate (%)	Q20 ^1^ (%)	Q30^2^ (%)	GC Content (%)
Specimen 1	350	54,836,979,600	99.98	45,063,446,400	0.03	95.93	90.81	45.03
Specimen 2	350	54,451,684,200	99.74	38,583,415,200	0.03	95.75	90.44	45.36

^1^ Q20: The ratio of data with accuracy above 99% in total data. ^2^ Q30: The ratio of data with accuracy above 99.9% in total data.

**Table 2:** Estimation of *S. sihama* (specimen 1) genome based on K-mer statistics.

Identity	K-mer	K-mer Depth	K-mer Number	Genome Size (Mbp)	Revised Genome Size (Mbp)	Heterozygous Ratio (%)	Repeat (%)
Specimen 1	17	70	36,648,430,961	523.55	508.50	0.92	21.25

**Table 3:** Statistics of *S. sihama* (specimen 1) assembled genome sequences.

	Identity	Total Length (bp)	Total Number	Max Length (bp)	N50 Length (bp)	N90 Length (bp)
Contig	Specimen 1	559,219,807	1,009,363	46,417	1362	171
Scaffold	Specimen 1	568,556,466	814,219	72,953	2173	219

**Table 4:** Simple Sequence Repeat (SSR) distribution statistics for *S. sihama* (specimen 1).

Statistics	Di-	Tri-	Tetra-	Penta-	Hexa-
SSR number	84,406	50,420	11,361	2200	870
Percentage	56.55%	33.78%	7.61%	1.47%	0.58%

## Supplementary Materials

The following are available online at https://www.mdpi.com/2076-2615/9/10/756/s1. Table S1: Top 5 similar species compared in the Nucleotide Sequence Database of NCBI. Table S2: Estimation of *S. sihama* (specimen 2) genome based on K-mer statistics. Table S3: Statistics of *S. sihama* (specimen 2) assembled genome sequences. Table S4: SSR distribution statistics of *S. sihama* (specimen 2). Figure S1: K-mer (k=17) analysis for estimation of the genome size of *S. sihama* (specimen 2). Figure S2: GC content and average sequencing depth of *S. sihama* (specimen 2) genome data used for assembly. Figure S3: Ratio of different SSRs in *S. sihama* (specimen 2).
