# Supplementary material for: A First Insight into a Draft Genome of Silver Sillago (Sillago sihama) via Genome Survey Sequencing
Source: Animals (Basel). 2019 Oct 1;9(10):756. doi: 10.3390/ani9100756 (PMC6827152; doi:10.3390/ani9100756)
Supplement: Supplementary file 1 [file animals-09-00756-s001.pdf]

Supplementary Materials:

Table S1. Top 5 similar species compared in the Nucleotide Sequence Database of NCBI.

Species	Similarity percentage (specimen-1 / specimen-2)
Dicentrarchus labrax	0.79 / 0.87
Haplochromis burtoni	0.34 / 0.39
Oreochromis niloticus	0.25 / 0.27
Takifugu rubripes	0.15 / 0.17
Haplochromis chilotes	0.12 / 0.12

Remark: Similarity percentage is proportion of got hit clean reads in random 5000 clean reads.

Table S2. Estimation of *S. sihama* (specimen-2) genome based on K-mer statistics.

Identity	K-mer	K-mer Depth	K-mer number	Genome Size (Mbp)	Revised Genome Size (Mbp)	Heterozygous Retio(%)	Repeat (%)
Specimen-2	17	59	31,380,732,374	531.88	514.98	0.93	21.18

Table S3. Statistics of *S. sihama* (specimen-2) assembled genome sequences

	Identity	Total length (bp)	Total number	Max length (bp)	N50 length (bp)	N90 length (bp)
contig	Specimen-2	555,167,043	987,613	36,911	1,342	178
scaffold	Specimen-2	562,570,762	811,241	68,155	2,072	220

Table S4. SSR distribution statistics of *S. sihama* (specimen-2).

Statistics	Di-	Tri-	Tetra-	Penta-	Hexa-
SSR number	71,123	48,622	10,889	2,114	702
Percentage	53.30%	36.43%	8.16%	1.58%	0.53%

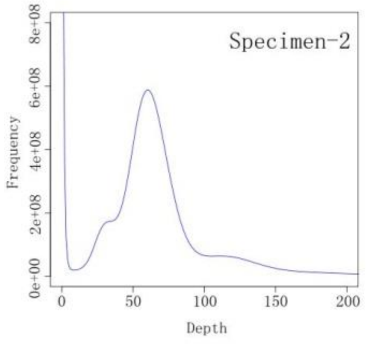

Fig. S1. K-mer (k=17) analysis for estimating the genome size of *S. sihama* (specimen-2).

Remark: The peak depth distribution of specimen-2 was at 59×; the estimated genome size was 531.88 Mb and the revised genome size was 514.98 Mb.

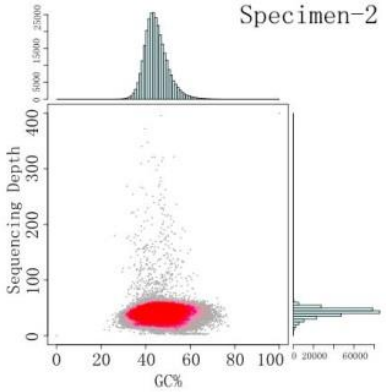

Fig. S2. GC content and average sequencing depth of *S. sihama* (specimen-2) genome data used for assembly. For the spot graphs,the x-axis is GC content and the y-axis is sequencing depth. For the bar graphs, the x-axis is sequencing depth distribution and the y-axis is GC content distribution.

Remark: Average GC content of specimen-2 was 44.99%.

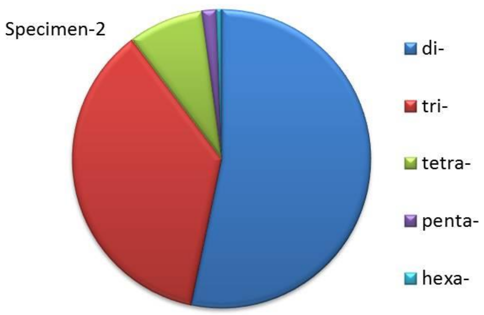

Fig. S3. Ratio of different SSRs in *S. sihama* (specimen-2).

Remark: Dinucleotide repeats were dominant (53.30%), followed by trinucleotide repeats (36.43%), tetranucleotides repeats (8.16%), pentanucleotide repeats (1.58%) and hexanucleotide repeats (0.53%).
